# 4-Meth­oxy­benzamidinium hydrogen oxalate monohydrate

**DOI:** 10.1107/S1600536812046351

**Published:** 2012-11-14

**Authors:** Simona Irrera, Gustavo Portalone

**Affiliations:** a Chemistry Department, "Sapienza" University of Rome, P.le A. Moro, 5, I-00185 Rome, Italy

## Abstract

The title hydrated salt, C_8_H_11_N_2_O^+^·C_2_HO_4_^−^·H_2_O, was synthesized by a reaction of 4-meth­oxy­benzamidine (4-amidino­anisole) and oxalic acid in water solution. In the cation, the amidinium group forms a dihedral angle of 15.60 (6)° with the mean plane of the benzene ring. In the crystal, each amidinium unit is bound to three acetate anions and one water mol­ecule by six distinct N—H⋯O hydrogen bonds. The ion pairs of the asymmetric unit are joined by two N—H⋯O hydrogen bonds into ionic dimers in which the carbonyl O atom of the semi-oxalate anion acts as a bifurcated acceptor, thus generating an *R* ^1^_2_(6) motif. These subunits are then joined through the remaining N—H⋯O hydrogen bonds to adjacent semi-oxalate anions into linear tetra­meric chains running approximately along the *b* axis. The structure is stabilized by N—H⋯O and O—H⋯O inter­molecular hydrogen bonds. The water mol­ecule plays an important role in the cohesion and the stability of the crystal structure being involved in three hydrogen bonds connecting two semi-oxalate anions as donor and a benzamidinium cation as acceptor.

## Related literature

For the biological and pharmacological relevance of benzamidine, see: Powers & Harper (1999 ▶). For structural analysis of proton-transfer adducts containing mol­ecules of biological inter­est, see: Portalone, (2011*a* ▶); Portalone & Irrera (2011 ▶). For supra­molecular association in proton-transfer adducts containing benzamidinium cations, see; Portalone (2010 ▶, 2011*b* ▶, 2012 ▶); Irrera *et al.* (2012 ▶); Irrera & Portalone (2012*a* ▶,*b* ▶,*c* ▶). For hydrogen-bond motifs, see Bernstein *et al.* (1995 ▶).
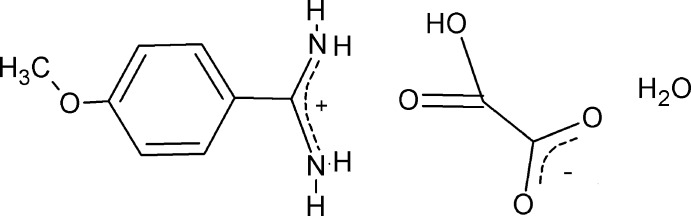


## Experimental

### 

#### Crystal data

C_8_H_11_N_2_O^+^·C_2_HO_4_^−^·H_2_O
*M* *_r_* = 258.23
Monoclinic, 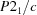

*a* = 7.1444 (8) Å
*b* = 9.0428 (7) Å
*c* = 18.115 (2) Å
β = 93.156 (10)°
*V* = 1168.5 (2) Å^3^
*Z* = 4
Mo *K*α radiation
μ = 0.12 mm^−1^
*T* = 298 K
0.18 × 0.12 × 0.09 mm

#### Data collection

Oxford Diffraction Xcalibur S CCD diffractometer
Absorption correction: multi-scan (*CrysAlis PRO*; Agilent, 2011 ▶) *T*_min_ = 0.978, *T*_max_ = 0.989
15203 measured reflections
2135 independent reflections
1693 reflections with *I* > 2σ(*I*)
*R*_int_ = 0.046

#### Refinement

*R*[*F* ^2^ > 2σ(*F* ^2^)] = 0.047
*wR*(*F* ^2^) = 0.110
*S* = 1.09
2135 reflections
201 parameters
2 restraints
H atoms treated by a mixture of independent and constrained refinement
Δρ_max_ = 0.16 e Å^−3^
Δρ_min_ = −0.15 e Å^−3^

### 

Data collection: *CrysAlis CCD* (Oxford Diffraction, 2006 ▶); cell refinement: *CrysAlis CCD*; data reduction: *CrysAlis RED* (Oxford Diffraction, 2006 ▶); program(s) used to solve structure: *SIR97* (Altomare *et al.*, 1999 ▶); program(s) used to refine structure: *SHELXL97* (Sheldrick, 2008 ▶); molecular graphics: *ORTEP-3* (Farrugia, 2012 ▶); software used to prepare material for publication: *WinGX* (Farrugia, 2012 ▶).

## Supplementary Material

Crystal structure: contains datablock(s) global, I. DOI: 10.1107/S1600536812046351/kp2440sup1.cif

Structure factors: contains datablock(s) I. DOI: 10.1107/S1600536812046351/kp2440Isup2.hkl

Additional supplementary materials:  crystallographic information; 3D view; checkCIF report

## Figures and Tables

**Table 1:** Hydrogen-bond geometry (Å, °)

*D*—H⋯*A*	*D*—H	H⋯*A*	*D*⋯*A*	*D*—H⋯*A*
N1—H1*A*⋯O3	0.92 (3)	2.43 (3)	3.180 (3)	138 (2)
N1—H1*B*⋯O2*W*	0.92 (2)	2.00 (2)	2.891 (3)	161 (2)
N1—H1*A*⋯O5^i^	0.92 (3)	2.37 (3)	3.096 (2)	135 (2)
N2—H2*A*⋯O3	0.86 (2)	2.05 (2)	2.869 (2)	159 (2)
N2—H2*A*⋯O4^ii^	0.86 (2)	2.34 (2)	2.827 (2)	116.4 (18)
N2—H2*B*⋯O6^ii^	0.88 (2)	2.09 (3)	2.932 (2)	159.5 (19)
O4—H4⋯O5^i^	1.02 (3)	1.56 (3)	2.5840 (19)	178 (2)
O2*W*—H21*W*⋯O5^iii^	0.85 (2)	2.15 (2)	2.976 (6)	163 (3)
O2*W*—H22*W*⋯O6^i^	0.88 (2)	1.97 (2)	2.853 (3)	177 (3)

Symmetry codes: (i) 
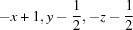
; (ii) 
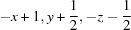
; (iii) 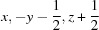.

**Fig. 1.**
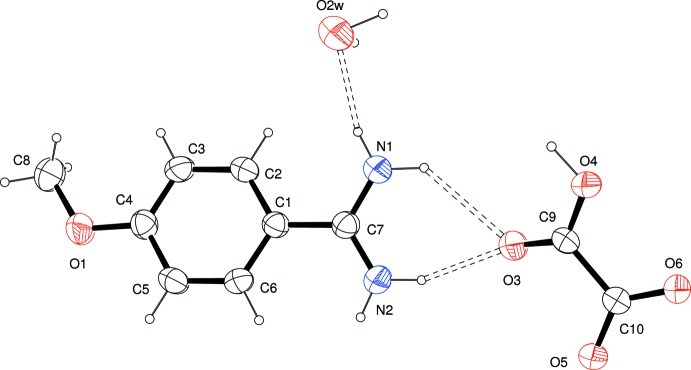
The asymmetric unit of (I), showing the atom-labelling scheme. Displacements ellipsoids are at the 50% probability level. The major occupied site of the disordered water molecule is shown, only. H atoms are shown as small spheres of arbitrary radii. Hydrogen bonding is indicated by dashed lines.

**Fig. 2.**
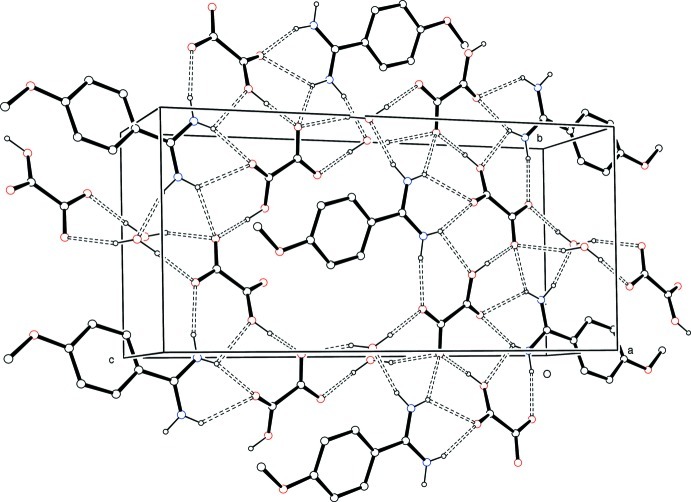
Crystal packing diagram for (I), viewed approximately down a. All atoms are shown as small spheres of arbitrary radii. For the sake of clarity, only the major occupied site of the disordered water molecule and H atoms involved in hydrogen bonding are shown. Hydrogen bonding is indicated by dashed lines.

## supplementary crystallographic information

### Comment

For some time now, we have studied proton-transfer adducts containing molecules of biological interest (Portalone, 2011*a*; Portalone & Irrera, 2011). In this context, benzamidine derivatives, which have shown strong biological and pharmacological activity (Powers & Harper, 1999), have been used in our group as bricks for supramolecular construction (Portalone, 2010, 2011*b*, 2012). Indeed, the bidentate hydrogen-bonding interaction between the amidinium and the carboxylate functional groups can be a powerful organizing force in solution and in the solid state.

The present study reports the single-crystal structure of the title molecular salt, 4-methoxybenzamidinium hydrogen oxalate monohydrate, (I), which was obtained by a reaction of 4-methoxybenzamidine (4-amidinoanisole) and oxalic acid in a water solution.

The asymmetric unit of the title compound comprises a non-planar 4-methoxybenzamidinium cation, a hydrogen oxalate anion and water molecule of crystallization (Fig. 1).

In the cation the amidinium group forms dihedral angle of 15.60 (6)° with the mean plane of the phenyl ring, which agrees with the values observed in protonated benzamidinium ions [14.4 (1) - 32.7 (1)°, Portalone, 2010, 2012; Irrera *et al.*, 2012)]. The lack of planarity in all these systems is obviously caused by steric hindrances between the H atoms of the aromatic ring and the amidine moiety. This conformation is rather common in benzamidinium-containing small-molecule crystal structures, with the only exception of benzamidinium diliturate, where the benzamidinium cation is planar (Portalone, 2010). Geometrical parameters of the 4-methoxybenzamidinium cation agree with those reported in previous investigations of other similar structures (Irrera *et al.*, 2012; Portalone, 2010, 2012; Irrera & Portalone, 2012*a*, 2012*b*, 2012*c*). In particular the amidinium group, true to one's expectations, features similar C—N bonds [1.317 (3) and 1.302 (2) Å], evidencing the delocalization of the π electrons and double-bond character.

The semi-oxalate anion is not planar, as the dihedral angle for the planes defined by the CO_2_H and CO_2_^-^ non-H atoms is 14.1 (3)°. Bond distances around atom C10 indicate a carboxylate group with delocalization of the negative charge between atoms O5 and O6. Bond distances around atom C9 are consistent with a carboxylic acid group.

In the crystal structure of (I), (Fig. 2), the hydrogen-bonding scheme is rather complex. Each amidinium unit is bound to three acetate anions and one water molecule by six distinct N—H···O intermolecular hydrogen bonds (N···O = 2.827 (2) - 3.180 (3) Å, Table 1) into a one-dimensional structure. The ion pairs of the asymmetric unit are joined by two N—H···O hydrogen bonds in ionic dimers, where the carbonyl atom O3 of the semi-oxalate anion acts as a bifurcated acceptor, thus generating an *R*^1^_2_(6) motif (Bernstein *et al.*, 1995). These subunits are then joined through the remaining N—H···O hydrogen bonds to adjacent semi-oxalate anions into linear tetrameric chains running approximately along crystallographic *b* axis.

Water molecule plays an important role in the cohesion and the stability of the crystal structure: they are involved in three hydrogen bonds connecting two semi-oxalate anions as donor (O2W—H21W···O5 and O2W—H22W···O6) and a benzamidinium cation as acceptor O2W···H1B—N1 (Table 1).

### Experimental

4-Methoxybenzamidine (0.01 mmol, Fluka at 96% purity) was dissolved without further purification in 6 mL of a hot aqueous solution of oxalic acid (0.01 mmol, Aldrich at 99.99% purity) and heated under reflux for 3 h. After cooling the solution to an ambient temperature, colourless crystals suitable for single-crystal X-ray diffraction separated from the solution after a week.

### Refinement

All H atoms were identified in difference Fourier maps, but for refinement all C-bound H atoms were placed in calculated positions, with C—H = 0.93 Å (phenyl) and 0.97 Å (methyl), and refined as riding on their carrier atoms. The *U*_iso_ values were kept equal to 1.2*U*_eq_(C, phenyl). and to 1.5*U*_eq_(C, methyl). Positional and thermal parameters of H atoms of the amidinium and the carboxylic groups were refined, giving N—H distances in the range 0.86 (2) - 0.92 (3) Å, and O—H distance equal to 1.02 (3) Å. The water molecule is disordered over two sites, O2W and O21W. Their occupancies were refined to to 0.85 (2) and 0.15 (2), respectively, by imposing that their values must add up to precisely one. The O—H distances of the H atoms attached to O2W were restrained to 0.85 (2) - 0.88 (2) Å.

### Crystal data

**Table:**

C_8_H_11_N_2_O^+^·C_2_HO_4_^−^·H_2_O	*F*(000) = 544
*M**_r_* = 258.23	*D*_x_ = 1.468 Mg m^−^^3^
Monoclinic, *P*2_1_/*c*	Mo *K*α radiation, λ = 0.71069 Å
Hall symbol: -P 2ybc	Cell parameters from 4235 reflections
*a* = 7.1444 (8) Å	θ = 3.0–29.1°
*b* = 9.0428 (7) Å	µ = 0.12 mm^−^^1^
*c* = 18.115 (2) Å	*T* = 298 K
β = 93.156 (10)°	Tablets, colourless
*V* = 1168.5 (2) Å^3^	0.18 × 0.12 × 0.09 mm
*Z* = 4	

### Data collection

**Table:**

Oxford Diffraction Xcalibur S CCD diffractometer	2135 independent reflections
Radiation source: Enhance (Mo) X-ray Source	1693 reflections with *I* > 2σ(*I*)
Graphite monochromator	*R*_int_ = 0.046
Detector resolution: 16.0696 pixels mm^-1^	θ_max_ = 25.4°, θ_min_ = 3.2°
ω and φ scans	*h* = −8→8
Absorption correction: multi-scan (*CrysAlis PRO*; Agilent, 2011)	*k* = −10→10
*T*_min_ = 0.978, *T*_max_ = 0.989	*l* = −21→21
15203 measured reflections	

### Refinement

**Table:**

Refinement on *F*^2^	Primary atom site location: structure-invariant direct methods
Least-squares matrix: full	Secondary atom site location: difference Fourier map
*R*[*F*^2^ > 2σ(*F*^2^)] = 0.047	Hydrogen site location: inferred from neighbouring sites
*wR*(*F*^2^) = 0.110	H atoms treated by a mixture of independent and constrained refinement
*S* = 1.09	*w* = 1/[σ^2^(*F*_o_^2^) + (0.0502*P*)^2^ + 0.2028*P*] where *P* = (*F*_o_^2^ + 2*F*_c_^2^)/3
2135 reflections	(Δ/σ)_max_ < 0.001
201 parameters	Δρ_max_ = 0.16 e Å^−^^3^
2 restraints	Δρ_min_ = −0.15 e Å^−^^3^

### Special details

**Table:**

Experimental. Absorption correction: CrysAlisPro, Agilent Technologies, Version 1.171.35.19 (release 27-10-2011 CrysAlis171 .NET) (compiled Oct 27 2011,15:02:11) Empirical absorption correction using spherical harmonics, implemented in SCALE3 ABSPACK scaling algorithm.
Geometry. All s.u.'s (except the s.u. in the dihedral angle between two l.s. planes) are estimated using the full covariance matrix. The cell s.u.'s are taken into account individually in the estimation of s.u.'s in distances, angles and torsion angles; correlations between s.u.'s in cell parameters are only used when they are defined by crystal symmetry. An approximate (isotropic) treatment of cell s.u.'s is used for estimating s.u.'s involving l.s. planes.
Refinement. Refinement of *F*^2^ against ALL reflections. The weighted *R*-factor *wR* and goodness of fit *S* are based on *F*^2^, conventional *R*-factors *R* are based on *F*, with *F* set to zero for negative *F*^2^. The threshold expression of *F*^2^ > 2σ(*F*^2^) is used only for calculating *R*-factors(gt) *etc*. and is not relevant to the choice of reflections for refinement. *R*-factors based on *F*^2^ are statistically about twice as large as those based on *F*, and *R*- factors based on ALL data will be even larger.

### Fractional atomic coordinates and isotropic or equivalent isotropic displacement parameters (Å^2^)

**Table:**

	*x*	*y*	*z*	*U*_iso_*/*U*_eq_	Occ. (<1)
O1	0.8980 (2)	0.07501 (16)	0.24710 (8)	0.0511 (4)	
N1	0.7104 (3)	−0.2554 (2)	−0.06019 (12)	0.0484 (5)	
H1A	0.671 (4)	−0.287 (3)	−0.1070 (16)	0.084 (9)*	
H1B	0.728 (3)	−0.326 (3)	−0.0238 (13)	0.060 (7)*	
N2	0.6975 (3)	−0.0163 (2)	−0.09613 (11)	0.0439 (5)	
H2A	0.668 (3)	−0.043 (2)	−0.1409 (14)	0.055 (7)*	
H2B	0.702 (3)	0.080 (3)	−0.0883 (12)	0.053 (7)*	
C1	0.7801 (2)	−0.0672 (2)	0.03205 (10)	0.0327 (4)	
C2	0.8530 (3)	−0.1659 (2)	0.08440 (11)	0.0420 (5)	
H2	0.8730	−0.2635	0.0707	0.050*	
C3	0.8970 (3)	−0.1231 (2)	0.15668 (11)	0.0414 (5)	
H3	0.9472	−0.1908	0.1910	0.050*	
C4	0.8654 (3)	0.0217 (2)	0.17730 (11)	0.0371 (5)	
C5	0.7954 (3)	0.1222 (2)	0.12537 (11)	0.0440 (5)	
H5	0.7761	0.2199	0.1390	0.053*	
C6	0.7543 (3)	0.0788 (2)	0.05387 (11)	0.0395 (5)	
H6	0.7085	0.1478	0.0194	0.047*	
C7	0.7288 (2)	−0.1139 (2)	−0.04408 (10)	0.0346 (5)	
C8	0.9800 (3)	−0.0223 (3)	0.30183 (12)	0.0542 (6)	
H8A	0.9075 (15)	−0.1129 (14)	0.3028 (6)	0.081*	
H8B	0.981 (2)	0.0250 (9)	0.3499 (7)	0.081*	
H8C	1.1076 (19)	−0.0452 (14)	0.2900 (5)	0.081*	
O3	0.6035 (2)	−0.17989 (15)	−0.22870 (8)	0.0506 (4)	
O4	0.4130 (2)	−0.35402 (14)	−0.27537 (8)	0.0448 (4)	
H4	0.454 (4)	−0.412 (3)	−0.2291 (15)	0.086 (8)*	
O5	0.4872 (2)	0.00180 (14)	−0.34351 (8)	0.0478 (4)	
O6	0.3608 (2)	−0.19541 (15)	−0.39997 (8)	0.0517 (4)	
C9	0.4936 (3)	−0.22516 (19)	−0.27658 (11)	0.0354 (5)	
C10	0.4403 (3)	−0.1325 (2)	−0.34652 (11)	0.0383 (5)	
O2W	0.7296 (9)	−0.5215 (3)	0.02842 (17)	0.0531 (13)	0.85 (2)
H21W	0.646 (3)	−0.530 (3)	0.0600 (14)	0.080*	
H22W	0.699 (4)	−0.578 (3)	−0.0102 (12)	0.080*	
O21W	0.845 (10)	−0.520 (2)	0.008 (2)	0.101 (17)	0.15 (2)

### Atomic displacement parameters (Å^2^)

**Table:**

	*U*^11^	*U*^22^	*U*^33^	*U*^12^	*U*^13^	*U*^23^
O1	0.0703 (10)	0.0445 (8)	0.0369 (8)	0.0073 (7)	−0.0118 (7)	−0.0061 (7)
N1	0.0728 (13)	0.0325 (10)	0.0385 (11)	−0.0018 (9)	−0.0083 (10)	−0.0023 (9)
N2	0.0607 (12)	0.0371 (11)	0.0328 (11)	−0.0035 (8)	−0.0074 (9)	0.0024 (8)
C1	0.0334 (10)	0.0303 (10)	0.0341 (11)	−0.0011 (8)	0.0007 (8)	−0.0002 (8)
C2	0.0576 (13)	0.0273 (10)	0.0406 (12)	0.0026 (9)	−0.0034 (10)	−0.0015 (9)
C3	0.0513 (13)	0.0332 (10)	0.0386 (12)	0.0015 (9)	−0.0080 (10)	0.0052 (9)
C4	0.0373 (11)	0.0383 (11)	0.0353 (11)	−0.0010 (8)	−0.0021 (8)	−0.0038 (9)
C5	0.0572 (13)	0.0292 (10)	0.0449 (13)	0.0061 (9)	−0.0045 (10)	−0.0041 (9)
C6	0.0494 (12)	0.0315 (10)	0.0370 (11)	0.0048 (9)	−0.0043 (9)	0.0042 (9)
C7	0.0354 (11)	0.0333 (10)	0.0348 (11)	−0.0016 (8)	−0.0007 (8)	0.0009 (9)
C8	0.0646 (15)	0.0563 (14)	0.0402 (13)	0.0030 (11)	−0.0105 (11)	0.0007 (11)
O3	0.0705 (10)	0.0363 (8)	0.0427 (9)	−0.0050 (7)	−0.0184 (8)	−0.0008 (6)
O4	0.0650 (10)	0.0293 (7)	0.0386 (9)	−0.0066 (6)	−0.0096 (7)	0.0046 (6)
O5	0.0765 (11)	0.0278 (7)	0.0379 (8)	−0.0054 (7)	−0.0081 (7)	0.0019 (6)
O6	0.0857 (11)	0.0329 (8)	0.0343 (9)	−0.0044 (7)	−0.0164 (8)	−0.0001 (6)
C9	0.0458 (12)	0.0257 (9)	0.0345 (11)	0.0045 (8)	−0.0006 (9)	−0.0040 (8)
C10	0.0511 (12)	0.0289 (10)	0.0346 (11)	0.0007 (9)	−0.0014 (9)	−0.0016 (8)
O2W	0.074 (3)	0.0401 (12)	0.0453 (15)	−0.0055 (13)	0.0009 (14)	−0.0065 (9)
O21W	0.15 (4)	0.067 (10)	0.078 (16)	0.022 (14)	−0.04 (2)	−0.021 (9)

### Geometric parameters (Å, º)

**Table:**

O1—C4	1.361 (2)	C5—C6	1.370 (3)
O1—C8	1.427 (2)	C5—H5	0.9300
N1—C7	1.317 (3)	C6—H6	0.9300
N1—H1A	0.92 (3)	C8—H8A	0.9696
N1—H1B	0.92 (2)	C8—H8B	0.9697
N2—C7	1.302 (2)	C8—H8C	0.9696
N2—H2A	0.86 (2)	O3—C9	1.209 (2)
N2—H2B	0.88 (2)	O4—C9	1.300 (2)
C1—C2	1.383 (3)	O4—H4	1.02 (3)
C1—C6	1.393 (3)	O5—C10	1.260 (2)
C1—C7	1.470 (3)	O6—C10	1.234 (2)
C2—C3	1.385 (3)	C9—C10	1.549 (3)
C2—H2	0.9300	O2W—O21W	0.92 (8)
C3—C4	1.383 (3)	O2W—H21W	0.854 (17)
C3—H3	0.9300	O2W—H22W	0.883 (17)
C4—C5	1.382 (3)	O21W—H22W	1.20 (6)
			
C4—O1—C8	118.00 (16)	C5—C6—H6	119.5
C7—N1—H1A	121.5 (18)	C1—C6—H6	119.5
C7—N1—H1B	120.6 (14)	N2—C7—N1	119.1 (2)
H1A—N1—H1B	118 (2)	N2—C7—C1	120.56 (18)
C7—N2—H2A	121.0 (15)	N1—C7—C1	120.28 (18)
C7—N2—H2B	123.3 (14)	O1—C8—H8A	109.5
H2A—N2—H2B	116 (2)	O1—C8—H8B	109.5
C2—C1—C6	117.90 (17)	H8A—C8—H8B	109.5
C2—C1—C7	121.50 (17)	O1—C8—H8C	109.5
C6—C1—C7	120.59 (17)	H8A—C8—H8C	109.5
C1—C2—C3	121.65 (18)	H8B—C8—H8C	109.5
C1—C2—H2	119.2	C9—O4—H4	111.6 (15)
C3—C2—H2	119.2	O3—C9—O4	124.20 (18)
C4—C3—C2	119.22 (18)	O3—C9—C10	121.61 (17)
C4—C3—H3	120.4	O4—C9—C10	114.18 (17)
C2—C3—H3	120.4	O6—C10—O5	126.03 (18)
O1—C4—C5	115.88 (17)	O6—C10—C9	118.28 (16)
O1—C4—C3	124.32 (18)	O5—C10—C9	115.68 (17)
C5—C4—C3	119.80 (18)	O21W—O2W—H21W	161 (2)
C6—C5—C4	120.39 (18)	O21W—O2W—H22W	83 (2)
C6—C5—H5	119.8	H21W—O2W—H22W	109 (3)
C4—C5—H5	119.8	O2W—O21W—H22W	47 (3)
C5—C6—C1	121.01 (18)		
			
C6—C1—C2—C3	0.9 (3)	C2—C1—C6—C5	−1.6 (3)
C7—C1—C2—C3	−178.30 (18)	C7—C1—C6—C5	177.63 (18)
C1—C2—C3—C4	0.7 (3)	C2—C1—C7—N2	−165.77 (19)
C8—O1—C4—C5	−176.59 (18)	C6—C1—C7—N2	15.0 (3)
C8—O1—C4—C3	3.7 (3)	C2—C1—C7—N1	15.5 (3)
C2—C3—C4—O1	177.98 (18)	C6—C1—C7—N1	−163.68 (19)
C2—C3—C4—C5	−1.7 (3)	O3—C9—C10—O6	164.7 (2)
O1—C4—C5—C6	−178.67 (17)	O4—C9—C10—O6	−14.1 (3)
C3—C4—C5—C6	1.1 (3)	O3—C9—C10—O5	−13.9 (3)
C4—C5—C6—C1	0.6 (3)	O4—C9—C10—O5	167.33 (17)

### Hydrogen-bond geometry (Å, º)

**Table:**

*D*—H···*A*	*D*—H	H···*A*	*D*···*A*	*D*—H···*A*
N1—H1*A*···O3	0.92 (3)	2.43 (3)	3.180 (3)	138 (2)
N1—H1*B*···O2*W*	0.92 (2)	2.00 (2)	2.891 (3)	161 (2)
N1—H1*A*···O5^i^	0.92 (3)	2.37 (3)	3.096 (2)	135 (2)
N2—H2*A*···O3	0.86 (2)	2.05 (2)	2.869 (2)	159 (2)
N2—H2*A*···O4^ii^	0.86 (2)	2.34 (2)	2.827 (2)	116.4 (18)
N2—H2*B*···O6^ii^	0.88 (2)	2.09 (3)	2.932 (2)	159.5 (19)
O4—H4···O5^i^	1.02 (3)	1.56 (3)	2.5840 (19)	178 (2)
O2*W*—H21*W*···O5^iii^	0.85 (2)	2.15 (2)	2.976 (6)	163 (3)
O2*W*—H22*W*···O6^i^	0.88 (2)	1.97 (2)	2.853 (3)	177 (3)

Symmetry codes: (i) −*x*+1, *y*−1/2, −*z*−1/2; (ii) −*x*+1, *y*+1/2, −*z*−1/2; (iii) *x*, −*y*−1/2, *z*+1/2.
